# Efficient fat suppression by slice-selection gradient reversal in twice-refocused diffusion encoding

**DOI:** 10.1002/mrm.21746

**Published:** 2008-11

**Authors:** Zoltan Nagy, Nikolaus Weiskopf

**Affiliations:** Wellcome Trust Centre for Neuroimaging at University College London (UCL), Institute of Neurology, University College LondonLondon, UK

**Keywords:** diffusion imaging, diffusion tensor imaging, fat suppression, chemical shift artifact, slice-selection gradient

## Abstract

Most diffusion imaging sequences rely on single-shot echo-planar imaging (EPI) for spatial encoding since it is the fastest acquisition available. However, it is sensitive to chemical-shift artifacts due to the low bandwidth in the phase-encoding direction, making fat suppression necessary. Often, spectral-selective RF pulses followed by gradient spoiling are used to selectively saturate the fat signal. This lengthens the acquisition time and increases the specific absorption rate (SAR). However, in pulse sequences that contain two slice-selective 180° refocusing pulses, the slice-selection gradient reversal (SSGR) method of fat suppression can be implemented; i.e., using slice-selection gradients of opposing polarity for the two refocusing pulses. We combined this method with the twice-refocused spin-echo sequence for diffusion encoding and tested its performance in both phantoms and in vivo. Unwanted fat signal was entirely suppressed with this method without affecting the water signal intensity or the slice profile.

The sensitivity of the MR signal to diffusion (1) can be exploited in imaging experiments (2) to study the microstructure of healthy (3,4), injured (5,6), or aging (7) tissue. However, because bulk movement of the entire head can create artifacts that manifest themselves as apparently excessive diffusion, and because the data collection takes a considerable amount of time, artifacts due to subject motion rendered the application of diffusion imaging difficult in vivo until the introduction of echo-planar imaging (EPI) methods (8).

Although EPI acquisition methods allow for the collection of all the data required for an entire two-dimensional (2D) image after a single excitation, one has to accept and deal with several drawbacks. Among these is the excessive sensitivity to chemical-shift artifacts; i.e., fat-shift, in the phase-encoding direction due to the comparatively low bandwidth in that orientation. Several methods have been developed to deal with this particular artifact. For example the short *T*_1_ inversion-recovery (STIR) method inserts an inversion pulse at the beginning, with the image acquisition initiated at the time when the fat has zero longitudinal magnetization. Alternatively, spectrally-selective RF pulses can be applied with a narrow bandwidth, centered on the frequency of protons in fatty tissue. The effect will be to nutate the lipid magnetization to the transverse plane while the water magnetization remains unaffected along the longitudinal axis. Using spoiler gradients, the fat signal is dephased prior to image acquisition. Combination of these two techniques is also possible, and known as spectral inversion-recovery (SPIR) (9,10). All of the above implementations for nulling the fat signal require additional RF and gradient pulses, thus increasing the specific absorption rate (SAR) and lengthening the acquisition time.

Another approach, the slice-selection gradient-reversal (SSGR) method (11), requires neither prepulses nor extra spoiling gradients, provided the sequence already contains two 180° refocusing pulses. The pivotal aspect of this method is to use slice-selection gradients of opposing polarity for the two refocusing pulses. Without fat saturation, the 90° pulse will excite both fat and water. However, it will excite fat in a slice that is slightly displaced relative to the slice of water, due to the chemical shift of fat (Fig. 1). Suppose that a positive gradient is used for the excitation pulse and the first 180° refocusing pulse. When the slice-selection gradient is reversed, the second 180° refocusing pulse will affect fat in a slice that is displaced in the opposite direction relative to the excited slice of water. In the final acquisition of the data, fat signal will only be refocused in and collected from the overlapping region that was exposed to both refocusing pulses in the center of the water slice.

**FIG. 1:**
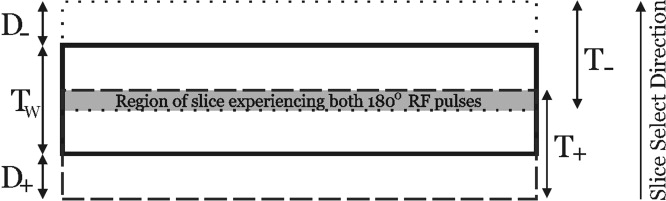
Illustration of the SSGR method of fat saturation. The solid rectangle depicts the position of the water slice with thickness T_w_. The 90° RF excitation pulse and the first 180° refocusing pulse both use a positive slice-selection gradient and excite fat in a slice that is slightly displaced from that of water (dashed line). The amount of displacement is denoted by D_+_. If the slice-selection gradient is reversed for the second 180° refocusing pulse, the excited slice of fat will be displaced in the opposite direction (dotted line). If the amplitude of the positive and negative slice-selection gradients are equivalent the displacement, D_–_ is identical in magnitude to D_+_ (i.e., D_+_ = D_–_ = D) and the thickness of the shifted slices (T_+_ and T_–_) remains constant. Under these conditions the thickness of fat that is excited and refocused along with the water is T_w_ – 2D. This region is the shaded area in the center of the water slice.

The extent of displacement of the fat slice relative to the water slice is

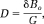
1
as given by Eq. [1] in the original work describing the SSGR method of fat suppression (11). Here, if δ is the chemical shift in parts per million (ppm), *B*_0_ is the main magnetic field in Tesla (T), and G is the slice-selection gradient strength in mT/m, then the units of D will be mm. From Fig. 1, it is also clear that the area that experiences both refocusing pulses is equal to T_w_-2D, where *T*_w_ is the nominal slice thickness of the excited water. For example, on our local machine the *B*_0_ field was 2.89T and the slice-selective gradient amplitude was 3.7 mT/m. With a relative chemical shift of 3.35 ppm for fat compared to water (12) the fat is displaced by 2.8 mm.

Note, however, that the SSGR method suppresses any off-resonance signal, not only those due to chemical shift. For instance, the field inhomogeneities that are present within the brain (13) shift the water signal frequencies as well.

Diffusion encoding is usually achieved by the introduction of large diffusion-encoding gradients (14). However, the employment of these large gradients leads to eddy currents in the machine, which can degrade the images. To reduce this effect, Reese et al. (15) developed the twice-refocused version of diffusion encoding, which contains four diffusion-encoding gradient lobes arranged around two 180° refocusing pulses. Thus, this sequence is ideally suited for implementing the SSGR method for fat suppression.

The aim of this study was to investigate whether the SSGR method would adequately suppress fat signal while maintaining image quality in diffusion-weighted images that were collected with the twice-refocused implementation of diffusion weighting. After careful quality assurance (QA), we conclude that this method of fat suppression performs well and can provide the extra benefit of reducing both the acquisition time and SAR.

## MATERIALS AND METHODS

All experiments were performed on a 3T whole-body scanner (Magnetom Tim Trio; Siemens Medical Systems, Erlangen, Germany) operated with a body transmit coil and a 12-channel head receive coil with the following general imaging parameters: TE = 90 ms, TR-per-slice = 300 ms, isotropic resolution = 2.3 mm, 60 axial slices, matrix size = 96 × 96, and field of view = 192 mm; with twice-refocused diffusion-encoding according to Reese et al (15).

### Phantom Experiments

**FIG. 2:**
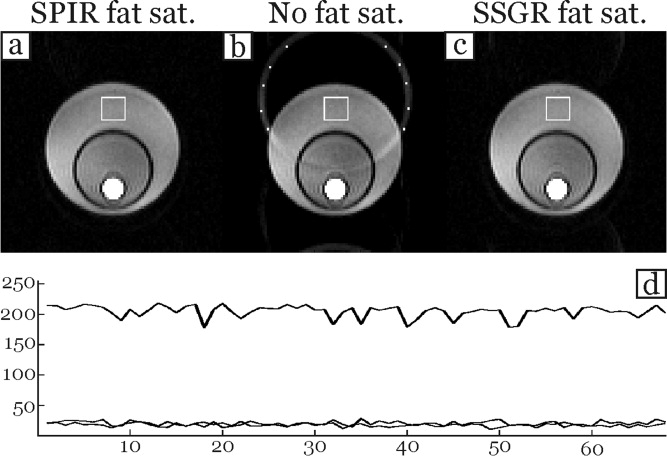
Comparison of the SSGR and the SPIR methods of fat suppression. Illustrative axial slice through the custom made phantom with an oil ring (phase encoding top-to-bottom). Acquisition with SPIR (**a**), without fat saturation (**b**) and with SSGR (**c**). All images (**a–c**) are windowed identically. The image in the middle demonstrates the need for some form of fat suppression. (**d**) The mean signal from the 10 voxels located in the shifted fat signal across the 68 acquired images (the first seven are reference images while the latter 61 are diffusion-weighted) for all three acquisitions.

Two different experiments were performed. The first experiment addressed the efficacy of the SSGR method at suppressing the unwanted fat signal. For this, a custom-made phantom was used, which contained four cylindrical compartments nested within each other. The outer ring was filled with sunflower oil, the middle two were filled with agarose gel and doped to mimic human white and gray matter in *T*_1_/*T*_2_-relaxation times, while the innermost cylinder contained distilled water (Fig. 2). This phantom was used to collect images both with and without fat suppression. When fat suppression was employed it was with either the SSGR method described above or a variant of the SPIR (10) method, as implemented in product sequences on Siemens scanners using only a 110° RF pulse and a shorter inversion time instead of a full 180° inversion.

The diffusion-weighted imaging scheme consisted of 68 images, each with a unique diffusion direction. The *b*-value was 100 s/mm^2^ for the first seven images. These are usually used as reference in the calculations of the apparent diffusion coefficient (ADC). The *b*-value was 1000 s/mm^2^ for the remaining 61 images. The latter 61 directions were uniformly distributed on the surface of a hemisphere, using the electrostatic minimization procedure (16). Instead of calculating the tensor from the data, we investigated the quality of fat suppression in the individual images.

The purpose of the second experiment was to investigate whether the slice profile was affected by the SSGR method. For this, another multipurpose phantom was used, built by Marconi Medical Systems, Inc. (Cleveland, OH, USA). It was filled with doped water and is used for general QA. In particular, it contained plastic wedges, which are useful for investigating slice profiles of 2D images (for details please see Ref.^10^; pages 212–214).

For the QA phantom measurements, apart from the reference image, only six diffusion-encoding directions were used, along the positive and negative principal gradient axes.

### In Vivo Experiments

A healthy adult volunteer was scanned twice, once without fat suppression and once using the SSGR method. In each case, the diffusion-weighting scheme with seven reference images and 61 noncollinear diffusion directions was used. Written informed consent was obtained prior to the experiment according to the guidelines of the local ethics committee.

### Image Postprocessing

All analyses of images were performed in the Matlab 7.0 (MathWorks Inc., Natick, MA, USA) environment.

To investigate how effective the two methods of fat suppression were, regions of interest (ROIs) were drawn on the images of the custom-made phantom, encompassing the shifted fat signal (Fig. 2b, white dots). The signal was extracted from corresponding ROIs in all 68 images of the other two acquisitions, which were fat suppressed either with the SPIR or SSGR method.

To investigate whether the slice profile was affected by manipulating the slice-selection gradient, a large square ROI was defined in the regions of the custom-made phantom that resembled the properties of white matter (Fig. 2a–c). The mean signal and the standard deviation (SD) within the ROIs were calculated for all three acquisitions: fat suppressed with either the SPIR or SSGR methods, and without fat suppression.

The scans of the QA phantom with the plastic wedges were used to measure the slice profile. From a slice close to the isocenter, a signal intensity profile through the plastic wedge was extracted from the images of the QA phantom. Note that this does not depict the slice profile itself; instead it indicates the pattern of the loss of signal where the image slice is interrupted by the plastic wedge; i.e., it is an indirect measure in which the slice profile is convolved with the wedge profile.

The sensitivity of the SSGR method to susceptibility-induced off-resonance artifacts was also investigated using the in vivo data. An inferior slice was taken from the reference images acquired with the SSGR method and without fat suppression. After realignment, the images were smoothed with a 10-mm full-width at half-maximum isotropic Gaussian kernel. Finally, the voxelwise ratio of the image intensity in the fat-suppressed image over that in the non-fat-suppressed image was calculated to indicate areas where the SSGR method affects the water signal.

## RESULTS

The three images at the top of Fig. 2 display the same center slice from the three different types of acquisitions performed on the custom made phantom: without fat suppression and with either the SPIR or the SSGR method. On visual inspection there was no discernable difference between the two fat suppression methods. While the fat signal was high in both the reference and diffusion weighted images without fat suppression, both the SPIR and SSGR fat-suppression methods eliminated the fat signal completely (Fig. 2d).

There was no indication that implementing the SSGR method caused a distortion of the slice profile. First, the signal extracted from the square ROIs (Fig. 2a–c) was not reduced by the SSGR method (without fat saturation: 100.2 ± 1.9 [mean ± SD]; SPIR = 101.7 ± 1.7; and SSGR = 105.9 ± 2.0), indicating that the excited and refocused volume is identical. Second, the results of experiments performed on the QA phantom with plastic wedges indicated that inverting the polarity of the slice-selection gradient for one of the 180° refocusing pulses had no effect on the thickness or profile of the slice. This convolution resulted in a voided signal across 13 voxels; therefore, the precision of the method is 2.3 mm/13 = 0.18 mm. The full-width at half-maximum of the curve representing the convolution of the slice profile with the plastic wedge was unchanged within this precision.

**FIG. 3:**
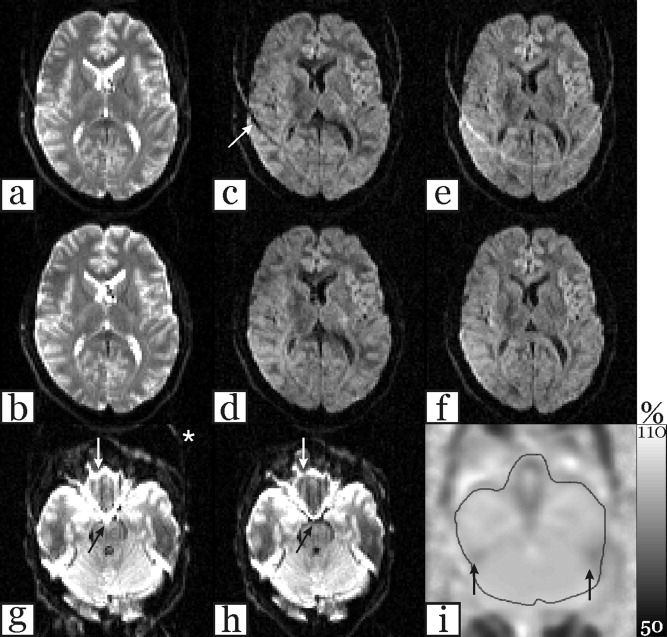
In vivo performance of the SSGR method of fat saturation. The top row displays images without fat suppression (**a,c,e**). The shifted fat signal is clearly visible on the reference image (a) and two illustrative diffusion-weighted images (**c,e**). Note how the fat interference can even cause a reduced signal level inside the brain (arrow in c). The middle row displays the corresponding images from an acquisition in which the SSGR method was used to suppress the fat signal (**b,d,f**). Images in (**a,b**) and in (**c–f**) are windowed identically. In the bottom row, inferior parts of the brain are displayed to demonstrate the interaction of the SSGR method of fat suppression with susceptibility-related off-resonance effects (**g,h,i**). (**g**) Acquisition without fat suppression (**h**) with SSGR fat suppression and (i) displays the ratio of the coregistered and smoothed versions of the images in (**h**) over (**g**). Low-intensity pixels in (**i**) indicate regions where the SSGR method of fat suppression reduced signal intensity. Note that regions which were most affected were also severely distorted by susceptibility artifacts (arrows).

The in vivo experiments confirmed that the fat signal is successfully suppressed by the SSGR method without negatively affecting the image quality (Fig. 3a–f). Furthermore, the SSGR method of fat suppression does not significantly exacerbate susceptibility-induced signal dropouts (Fig. 3g–i). However, in some small areas suffering from severe susceptibility-related frequency offsets; e.g., the medial orbitofrontal cortex as well as the temporal lobes around the ear canals, the signal amplitude was reduced (arrows in Fig. 3g–i) due to the inadvertent suppression of the water off-resonance signal.

## DISCUSSION

EPI images suffer from fat-shift artifacts as well as eddy-current distortions (17). The latter is especially troublesome in diffusion-weighted images. A pulse sequence can be implemented that deals with both of these problems efficiently. Reese et al. (15) developed a twice-refocused spin-echo method that reduces eddy-current effects; i.e., they used two 180° refocusing pulses which are straddled by four diffusion-encoding gradient lobes. Also using two 180° refocusing pulses, Gomori et al. (11) reported on the SSGR method of fat suppression, which uses slice-selection gradients of opposite polarity for the two 180° pulses, taking advantage of the chemical shift of fat with respect to water. We combined these two methods for an eddy-current compensated diffusion tensor imaging (DTI) EPI sequence with efficient fat suppression.

There are several advantages of using the SSGR method of fat suppression. First, it does not increase acquisition time as do STIR and SPIR (12). Second, because additional RF pulses are not needed, the SAR is reduced compared to STIR and SPIR (12). Finally, diffusion scans are often long, with a high-duty cycle, which can lead to drifts of the center frequency of the magnet over the experiment (18,19). This causes both the fat and the water signal to shift in frequency, making the SPIR fat suppression less effective and possibly suppressing the water signal. The SSGR method, however, is rather insensitive to this problem.

If the distance, D, by which the fat slice is excited away from the water slice is at least one-half of the nominal slice thickness, the fat signal is suppressed entirely (Fig. 1). To accurately estimate the value of D, and in turn the effectiveness of the method, one must know the exact value of *B*_0_ and the slice-select gradient amplitude. For our setup, the *B*_0_ was 2.89T and the slice-selection gradient amplitude was 3.7 mT/m. For the chemical shift, δ, for fat of 3.35 ppm relative to water (12) the fat slice displacement was 2.8 mm. This value for D is more than adequate because the slice thickness was only 2.3 mm. It is worth noting that Eq. [1 also shows that the method performs better for higher magnetic fields and weaker slice-selection gradients. However, the equation does not explicitly include the bandwidth of the RF pulses. For example, a weaker slice-select gradient, in conjunction with a lower bandwidth RF pulse, can be used to improve the fat suppression while achieving the same slice thickness at lower magnetic fields.

Consideration must be given to all off-resonance effects, not only chemical shift, since they have the potential to reduce the signal intensity. In this respect, susceptibility-induced field inhomogeneities (13) in the brain pose a problem. On our local 3T scanner, the field inhomogeneities within human brains ranged between –70 Hz and 120 Hz (determined by field mapping (13)). This is a relative frequency shift of up to 1 ppm, which is less than one-third of the 3.35 ppm (12) chemical shift of fat. In the worst case, this results in a slice that is excited approximately 0.82 mm away from the respective on-resonance water slice (see Eq. [1). With a slice thickness of 2.3 mm, the portion of the slice that is refocused, having experienced both RF pulses, is 2.3 mm – (2 × 0.82 mm) ≈ 0.7 mm. In other words, the signal in these areas may be decreased by up 69%. However, the regions of the human brain suffering from such severe field inhomogeneities are usually excluded from further analysis in DTI studies due to the severe distortions.

For situations in which the susceptibility artifacts are moderate but the signal loss is of concern, the extent of the signal loss can be reduced by increasing the amplitude of the slice-selection gradient, along with the bandwidth of the RF excitation pulse (see Eq. [1]).

It should be noted that off-resonance effects can also affect the SPIR implementation of fat suppression, and reduce the signal intensity, if the frequency of the off-resonant water signal falls within the bandwidth of the fat-selective RF pulse (20).

## CONCLUSIONS

The SSGR method of fat suppression is readily incorporated into pulse sequences that use the twice-refocused implementation of diffusion encoding. This method can completely suppress the fat signal and it provides the added benefit of reducing acquisition time and the SAR compared to the standard SPIR fat suppression. The reduction in SAR is particularly beneficial at high field strengths at which SAR can be a limiting factor in imaging experiments.
